# Sexual and Reproductive Health in Women with Pulmonary Hypertension: A Qualitative Study

**DOI:** 10.1007/s10508-022-02284-w

**Published:** 2022-02-14

**Authors:** Sabrina Cipolletta, Giorgia Ravasio, Maurizio Bussotti

**Affiliations:** 1 Department of General Psychology, University of Padua, Via Venezia 8, 35131 Padua, Italy; 2 Cardiology Rehabilitative Unit, IRCCS, Maugeri Clinical Scientific Institutes, Milan, Italy

**Keywords:** Pulmonary hypertension, Pregnancy, Qualitative research, Reproductive health, Sexuality

## Abstract

Pulmonary arterial hypertension (PAH) is a devastating disease characterized by elevation in pulmonary artery pressure causing progressive symptoms: shortness of breath, fatigue, and a decline in functional ability. Research on the impact of PAH on sexual and reproductive health was sparse. The aim of this study is to explore sexual and reproductive health of women with PAH in relation to their illness experience. Twenty-five women with PAH participated in semistructured interviews. A thematic analysis was conducted on the transcripts using the ATLAS.ti software. Four main themes were identified: illness experience, intimate relationship, sexuality, and attitudes toward pregnancy. Results showed that illness changed women’s self-perception, couples’ relationship, sexuality, and the idea of an eventual pregnancy. The relationship with the partner was usually indicated as an important resource, whereas communication with health care professionals was a critical but also potential key resource for the future. Results point to the need for intervention strategies to support women with PAH and help them make aware choices. Moreover, intervention strategies may inform health care interventions and policies for tackling the challenges posed by this illness.

## Introduction

Pulmonary arterial hypertension (PAH) is a very serious rare disease that affects the vessels of the lungs, causing a significant increase in the resistance of pulmonary circulation, an increase in blood pressure at the level of the pulmonary artery and, consequently, a decompensation of the right side of the heart. It comes in various forms (familial, related to congenital heart disease, secondary to autoimmune diseases or liver diseases, idiopathic) and various functional classes in order of severity. The most frequent symptoms are shortness of breath and rapid fatigue even during the simplest activities. Existing treatments can help to stabilize the condition, prevent and treat flare-ups, and improve patients’ quality of life but mortality from PAH still remains high (Delcroix & Howard, 2015; Galié et al., 2016). The prevalence of PAH is estimated at 15–50 people per million with a female-male ratio of 2.41 (Beshay et al., 2020; Ge et al., 2020).

Sexual and reproductive health may be strongly affected by PAH for several reasons. First, patients fear experiencing breathlessness and fatigue during sex (Collins et al., 2012; Hill et al., 2018; Kaptein et al., 2008). Second, pumps, catheters, and skin sites required for the continuous infusion of therapies may pose challenges to engaging in sexual behaviors and cause a negative body image (Hill et al., 2018). Third, as in the case of heart attacks (Schmitz & Finkelstein, 2010), the disease takes over the patients’ lives and there is no time or energy left to devote to the sexual sphere. The Pulmonary Hypertension Association of American and European patients and caregivers found that approximately 75% of patients had difficulty being fully intimate with their partners and 20% reported this as their primary concern (Studer et al., 2012). Correspondingly, 72% of caregivers who are partners of patients with PAH reported a decrease in sexual relationships (Guillevin et al., 2013). Caregivers feel less close to their spouse (23%) and feel that their spouse sees them more as a caregiver than as a lover (18%). In addition, with PAH, fears of pregnancy may impact sexual and reproductive health in women of childbearing age and their partners because pregnancy is strongly discouraged, due to the risk of pregnancy-related death (Hemnes et al., 2015; Pieper et al., 2014), whereas counseling on contraception is strongly recommended (Hill et al., 2018; Thorne et al., 2006). Therefore, healthcare workers and researchers should pay attention to the sexual health of people with PAH.

A common assumption among both patients and physicians is to consider sexuality as being confined purely too physical acts, but achieving orgasm is only one aspect. Our sexuality represents who we are and how we feel toward ourselves, but also toward those around us (Langfeldt & Porter, 1986). The World Health Organization (2002) defined sexual health as “a state of physical, emotional, mental and social well-being in relation to sexuality; it is not merely the absence of disorder, dysfunction or infirmity. Sexual health requires a positive and respectful approach, including the ability to have safe and pleasurable sexual experiences.” Although sexual health can be considered as an integral part of one’s quality of life and relationship, it has been little investigated from this perspective. The focus is usually on sexual disfunction, unwanted pregnancies, and other topics that are of fundamental importance, but which primarily focus on negative experiences and tend to structure sexual practices as risky and difficult to manage, leading to a segmentation of sexuality. On the contrary, the positive sexuality framework (Williams et al., 2015) focuses on the importance of sexual pleasure, choice, and diversity, thus underlining the uniqueness of sexual experience. In fact, sexuality varies throughout life, especially when considering illness or disability (Carpenter, 2010).

To date, only one study has specifically investigated the sexual health of women with PAH (Banerjee et al., 2018). This found that they have high levels of sexual distress that impact their overall quality of life. The results of this study bring to the fore the need for a more comprehensive understanding of the experiences of women with PAH related to sexual health. The aim of the present study was to qualitatively explore these women’s sexual and reproductive health in relation to their illness experience. The exploratory nature of this study and the limited existent literature make qualitative research the most suitable method to investigate such delicate and intimate topics as sexuality and pregnancy, allowing a deeper understanding of the situation (Hsieh & Shannon, 2005).

## Method

### Participants

Twenty-five women aged between 22 and 71 years (*M* = 47.36 ± 11.79 years old) participated in the study. The participants’ sociodemographic and clinical data are reported in Table 1. The inclusion criteria were (1) diagnosis of pulmonary arterial hypertension, (2) identifying as a woman, and (3) understanding the Italian language and being able to interact with the interviewer. The inclusion criteria were developed to attain the best possible heterogeneity of the sample in line with “theoretical sampling” (Flick, 2009). This method makes it possible to explore the phenomenon in different situations and to choose and enlarge them on the basis of the hypotheses; new situations or cases may be involved according to the new hypotheses to be tested, thereby data collection and analysis of the interviews proceed simultaneously. Our sampling ended once we considered that theoretical saturation had been reached; that is, when themes started to become repetitive, thus suggesting that gathering more data would yield no further theoretical insights about the emerging theory.

**Table 1:** Participants’ characteristics (*N* = 25)

	N
Illness severity (functional class)	
I	5
II	8
III	9
IV	3
Years of illness	
Mean (SD)	10,29 (± 11,44)
Nationality	
Italian	24
Philippines	1
Education level	
Mean years (SD)	12,08 (± 3,49)
Employment	
Unemployed	13
Employee	9
Student	1
Freelance	2
Children	
No	15
Before the diagnosis	9
Adopted	1
Partner	
Yes	20
No	5

All the participants were in the care of the Maugeri Clinical Scientific Institutes, in Milan (Italy). The hospital psychologist contacted participants by telephone to ask them whether they wanted to take part in the research, presenting the aims and methods and specifying that the participation in the study was voluntary and fully independent from the care received at the Institute. Only two participants declined the invitation to participate in the study because they had no time available for the interview. Individual appointments were set with those who had shown interest and were available for interviews. A different psychologist, who was trained to perform semi-structured interviews, conducted the interviews.

### Measures and Procedure

Semi-structured interviews (Kvale & Brinkman, 2009) were carried out between October 2019 and November 2020. Although conversational and flexible, the interviews followed a common structure of open-ended questions, which were created ad hoc by the researchers based on existing literature and field testing with a potential participant to discuss the track of the interview and address the questions to elicit a participant’s first-hand experience (Kallio et al., 2016).

Each interview started with the collection of the women’s personal data and went on to explore disease information (What is your experience of PAH? When and how were you notified of the diagnosis? What were your initial reactions? Since diagnosis/onset, how has your life been over time?), sexual and emotional health (Have there been any changes in the relationship with your partner and/or in your sexuality? Do PAH symptoms affect desire and the sexual act? Has having a disease such as this affected your femininity? Did you receive information about sexuality from medical personnel? Did you bring up the topic yourself? Are you satisfied with the answer? Did you make use of other sources [e.g., internet, friends, or disease associations]? Has religion played a role in your sexual experience? Do you use any contraceptive methods?), pregnancy (Would you like to have children? Have you been informed about the risks of pregnancy? What was your reaction and that of your partner? Did you talk about it? Have you ever had an abortion?).

Twelve interviews were conducted in an individual setting in a quiet room in a clinical psychology office at the Maugeri Clinical Scientific Institutes. However, due to the restrictions imposed to contain the COVID-19 contagion, the following 13 interviews were performed via a video call and the participants were encouraged to find a quiet place that could guarantee confidentiality. The interviews were administered by a clinical psychologist trained in performing research interviews. Interviews lasted about 60 min and were recorded, as well as transcribed, verbatim.

### Data Analysis

The qualitative analysis followed the five steps that Braun and Clarke (2006) suggested for thematic analysis. The ATLAS.ti8 software was utilized to manage the data. Initially, it was necessary to familiarize ourselves with the interviews’ data, reading them repeatedly and actively (taking notes and gaining a general idea of the data collected). Subsequently, two coders independently generated initial codes from the first interviews to identify pieces of information that were similar to each other. These were compared with those found in the other interviews, identifying differences and overlaps. The codes were then allocated to potential themes that were reviewed and named. A comparison between the themes generated by each coder showed only a few discrepancies, which were resolved by discussion to develop a shared codebook. One of the researchers applied the final codebook to analyze the interviews and the second researcher supervised the analysis and checked if there were discrepancies between their analysis of certain quotations and the first researcher’s analysis. In these cases, the researchers arrived at a shared decision through discussion and adjusted the codebook if needed. Both the coders are psychologists. They discussed the final codebook with the third author, who is a physician and could offer a different perspective on the analysis.

One of the key characteristics of thematic analysis is represented by the high degree of flexibility. Because there are no predetermined categories, each theme is directly derived from the participants’ stories through a deep analysis of the interviews, managing to enclose numerous details within it (Hsieh & Shannon, 2005). The themes can be identified by predominance or importance; not only for how often they appear within the text, but also based on the judgment of the investigator (predominance), or that they must contain important information (importance). Furthermore, to be considered, the themes must possess internal homogeneity and external heterogeneity (Braun & Clarke, 2006).

The study was conducted according to the Consolidated Criteria for Reporting Qualitative Research checklist (Tong et al., 2007). Coherence and reliability were achieved by accurately reporting the interviews’ administration, data analysis, and consistent use of each interview’s quotations. Reflexivity was sought through repeated comparison of the themes with regard to the data, and discussions were held between the researchers about alternative interpretations of the results (Yardley, 2000).

## Results

Four main themes were identified: illness experience, intimate relationships, sexuality, and attitudes toward pregnancy. Table 2 reports the codes contained in each theme with the respective frequencies. To contextualize and support the themes, many quotations translated from Italian are used. To respect the participants’ anonymity, quotations are identified by pseudonyms.

**Table 2:** Themes and codes with the number of interviews where each code was found in parenthesis

Themes	Codes
Illness experience	Communication of the diagnosis experienced negatively (4) Initial negative reactions (24) Difficulties of living with the disease (25) Changes in the sense of femininity and beauty (18) Pervasive disease (6) Disease as a fixed though (8) Internet as a negative source of info (13) Support of WhatsApp group (6)
Intimate relationship	Avoidance of specific situations (2) End of relationships (3) People do not understand the disease (9) More understanding and attentive partner (15) Excessive concern on the part of other people (4)
Sexuality	Lack of information (16) Physical difficulties (9) Thoughts and limitations due to the disease (25) Fear of pregnancy (2) Sexuality improved over time (15) Religiosity (1)
Attitudes toward pregnancy	Negative communication of the risks of pregnancy (4) Feelings of injustice (16) Adoption (8) Hope that the situation will change (4) Non-acceptance of the impossibility of parenthood (5) Responsible partner (9) Feeling lucky to have had children before the disease (7)

### Illness Experience

Participants reported the disease as something unexpected that suddenly upset their lives. Initial reactions related to the communication of the diagnosis were varied. Some participants reported feeling lost and worried, others were angry or in shock, and others still were sad and frightened by the uncertainty inevitably associated with the disease (being a rare disease, participants often had no idea what it meant to have PAH). After receiving the diagnosis and the subsequent impending hospitalization, only one participant, feeling exhausted, claimed to have been almost relieved at the news of impending hospitalization.

At the beginning you do not realize what is happening because all happens so suddenly. (Giada, 49 yrs)

At the beginning I reacted very badly in the sense that I felt I had been beaten because I did not expect it; at 45 years old you are still young, you would never think about something like that. (Caterina, 47 yrs)

To be very honest, for me, it has been the need to rest, not so much as to recognize the illness as the need to stop … I used to work for eight hours per day in a company where I was used as a joker …. I stopped and I started to say to myself that maybe I needed to stop as a worker, I really need to stop, I was tired, I was really tired. (Paola, 50 yrs)

Associated with the illness were narratives of feeling ashamed of both their changing bodies and the limitations imposed by the disease, but also, in general, the shame of being ill. Some participants complained that other people seemed not to understand the situation or even belittle it: “I just never talked about it. If someone asked me … I was ashamed … I felt that I was not like I was before. Now, I had to accept this thing: I am sick” (Giada, 49 yrs). Another participant stated:

For example, the relatives, not from my side but from my husband’s side, always say, “No it’s not true, you’re crazy. This is a trifle. It’s nothing.” Yes, at first, I was angry, then I said what do I care? Think what you want. (Barbara, 53 yrs)

The communication of the diagnosis represented a very delicate moment. Most participants reported receiving calm and human communication. For others, the communication was cold and careless: “The head of cardiology was unforgettable, because he was very cold, not caring about how I was feeling …. ‘It’s progressive, and then it can lead to death,’ he said. I looked at him: ‘Are you kidding me?’” (Anna, 49 yrs).

Most of the women said that they searched the internet about their disease and came across discouraging and negative information. The support of a WhatsApp group, initially created with the help of the psychologist at the hospital, was considered a valid means of obtaining help with the initial fear and disorientation.

Participants described the illness as pervasive in their lives and limiting their freedom in many ways, such as not making a physical effort; organizing travel and journeys, taking into account medicines, their storage, and, in the case of oxygen, cylinders; methods of transport; and avoiding excessively long journeys or high-altitude destinations. For this reason, some participants felt forced to reorganize their lives according to their new needs, learning to respect their limits:

So, I have to organize myself a little more about the commitments I have, and especially about my physical and psychological state, because then I know that if I always take this point to the limit, I feel more fatigued, more tired. (Sara, 24 yrs)

Although an attempt to go back to normality is often reported, the illness still represents a fixed thought that cannot be avoided, and that led some participants to consider PAH as a traveling companion with whom they have to share their life:

There’s something different about me that I can’t explain. Don’t ask me, because I can’t explain it. … I don’t know how to express it. There is something that marks you. I don’t know how to say … the disease is present in your life, isn’t it? Even if you stay positive and try to do everything you did before, the disease is present. It’s as if you have a life companion now. You are different perhaps in this sense. (Valentina, 50 yrs)

Participants’ sense of femininity and beauty also underwent changes due to the illness: scars and external medications (e.g., an infusion pump) compromised the participants’ body image, causing discomfort in showing their bodies. Nevertheless, they each experienced this in varying ways. Some completely lost their sense of femininity and considered body care an additional commitment to add to the efforts accumulated during the day, as well as a waste of time. Others could regain their wounded femininity by giving it a new meaning, which could only be done by taking into account the changes that the illness caused in the participants’ lives:

So, I prioritize myself. I discard the things that make me tired. Makeup and dieting is tiring because you have to give up things, and I say, why do I have to give it up? What do I care about it? I like something and I do it. I like to drink a beer and I drink it. What do I care? It is useless to give up [something you like]. I mean, at this point I say, why should I give it up? I enjoy it and that’s it, and this is a change; yes, before I was one who cared, put on makeup. So … now I don’t care. (Barbara, 53 yrs)

### Intimate Relationships

The awareness of physical impediments and other problems implied by PAH led some of the participants to avoid specific situations in which they could meet new people and establish an intimate relationship:

I try not to have opportunities to meet people. I stay in my circle of people, and I don’t look for opportunities to meet anyone. I mean, I really don’t think about it. I set it aside because you have to find a person who is empathetic about this, which is not easy. It has been my baggage for 40 years and will still be my baggage for the future years. So, it is not a simple thing. That is, I would almost need a caregiver, putting it in a proper way. It’s not that simple. (Maria, 40 yrs)

Other participants experienced the end of their intimate relationships and attributed this to the partners because they were not able to understand PAH and its complications:

I went on dates with two men who told me this [that the problem was the illness]. I say, ‘Well then, the problem is you, not me.’ I mean, I don’t think that the problem is the disease. I think that some people are not able to deal with the disease; let’s put it that way. (Sara, 24 yrs)

The women who remained with a steady partner recounted that the partner became sweeter, more protective, attentive, and caring, and supported them during the various therapies and in daily life, making them feel loved despite everything. The partner’s fear about the woman (e.g., that she does not tire too much or that she is well) was also a recurring theme in the stories of these women: “He’s always been there. In fact, he surprised me because I said that he could run away, could he not? And so, on an emotional level, I received huge personal recognition” (Anna, 49 yrs). Despite this, sometimes these women felt bothered and overwhelmed by the excessive concerns of other people, especially their partners.

He is a bit heavy because he says, “I do not want that you take the train” and effectively I had been really bad but sometimes he seems a little excessive to me. (Giada, 50 yrs)

### Sexuality

None of the participants received information regarding sexuality and it was mentioned only in relation to the risks of pregnancy. Shame was often associated with sexuality and prevented participants from openly speaking with their doctors about this topic, especially if the doctors were male. Neither did the participants seek out information from other sources. Other participants did not speak about this topic because they avoided thinking about it: “I’m so overwhelmed with other things. I don’t … I probably don’t think about it …. I have different priorities. I have things that come before that, so it probably took a back seat” (Maria, 40 yrs).

You know these things are not things you can always talk about with everyone. With the doctors, let’s say, I don’t like to talk about it. I only talk about it with female doctors, but my doctor is male …. In fact, when I say, “Doctor, I can’t tell you …” and he says, “Ah, that’s fine,” he understands that I prefer to talk about it with the female doctor. (Daniela, 42 yrs)

Participants described tiredness and fatigue as some of the most pervasive symptoms of PAH that limited their sexual activity and intimacy. The use of oxygen cylinders or subcutaneous infusion pumps connected to an external body (usually contained in a small bag) to infuse medications without interruption represented an additional obstacle:

Well, I couldn’t breathe, I was attached to oxygen. So, you just can’t do it, and then maybe, if you’re meant to keep on oxygen, you’ll adapt to that, too, but I couldn’t do it. So, the first times, the first months were just a, stop, that’s it, let’s see what will happen. (Valentina, 50 yrs)

The all-consuming thought of the disease and its limitations led participants to think about it even in the most intimate moments. The fear that their partner might physically harm them in some way, or the fear of becoming pregnant despite the use of contraceptives, affected participants’ and their partners’ sexual experience: “He is afraid of harming me, thereby there is an approach like if we were brother and sister or almost” (Giovanna, 34 yrs).

The fear of saying, ‘I’m over you. I’ll crush you, maybe I’ll cause you problems.’ This caused us a bit of fear. … Also, when there was the machine, because there’s a whole thread, there’s a whole thing to manage. (Paola, 50 yrs)

There is always the fear of getting pregnant, therefore we have always used contraceptives … I did not feel secure and probably neither he did. However, there was the desire to stay together but with the fear that if it [getting pregnant] would happen, oh my god, what will happen? What shall we do? (Chiara, 37 yrs)

However, over time, the participants’ sexuality generally improved and was no longer a problem:

But the impact was there in the physical sense at the beginning. … Then, there is the psychological side. Then, slowly you come back to your normal sexual life. Everything is normal again after the moment of adaptation. (P3, 50 yrs, FC III)

The participants did not describe any impacts of contraception on the quality of their sexual relationships, either because they complied with their medical prescriptions without questioning them, or there were other conditions that prevented them from becoming pregnant (e.g., menopause or uterine removal). Only one participant would have preferred to keep contraception aside from her sexual relationship because her religiosity led her to consider sexuality as linked to pregnancy.

They [physicians] told me to be careful because I cannot take the pill because I am taking anticoagulants together with other things. Let’s say that I cannot use any other contraception but condoms. Consequently, I must use them and that’s all. (Beatrice, 44 yrs)

Before we used natural [contraception] methods in the sense of on the basis of my periods. We also had unprotected intercourse, something that we still do. But now there is some fear because you are not so mathematically sure, or [it is] better if you do not know these methods well and if you are not careful there is a risk. Therefore, yes, the religion, my husband and I would have preferred not to use any contraception even because, of course, this changes the affectivity and emotivity of the intercourse. (Chiara, 37 yrs)

Although other participants also considered themselves to be believers, specifically Catholics, religion played a role in sexuality only for this woman, who perceived a loss of meaning of the sexual act as a consequence of the impossibility of having a child and the fear of getting pregnant:

I have never read anywhere that Jesus said that we do not have to have sex. This is something that someone else has added and that, today, thank God, is totally clear. Thereby, [religion] has had an important role in many other things [in my life] but not in sexuality. (Valentina, 50 yrs)

We never had the peace of mind to say, “Well if it happens, it’s okay, we welcome it,” and this gave me a lot of pain at the beginning. I cried, because I wanted our love to turn into a deed and that the deed would turn into something splendid like a child. Maybe, also because of a religious issue, an act that generates love that is, born from love can generate other love that can be a child. (Chiara, 37 yrs)

### Attitudes Toward Pregnancy

Most participants reported having received information regarding the contra-indication for pregnancy. In some cases, this was communicated gently and sensitively, but in others, participants perceived the communication as too hasty to allow them to accept the news:

I experienced it as something that had been taken away from me. It’s as if they had cut off my leg but no one had told me anything. They left me there and said, “Well now, go on, go, nothing happened.” I did not experience the communication in a delicate way. In my opinion, communication should be made with more delicacy and time …. It is as if they had really taken away an important part of me, but they didn’t give it the right importance, the right weight. In such an unfair way, they took it away from me, and then they acted as if nothing happened. (Chiara, 37 yrs)

Participants described the moment of the discovery of the risks of pregnancy as an injustice and associated feelings of anger, regret, and frustration for not having the possibility of living the “magic moment” of pregnancy and having children with their partner. In one case, the lack of a child conveyed a feeling of an incomplete family and a lack in the woman’s personal role.

Pregnancy is a huge scar. It stays with you, and every now and then it comes out. It is a scar that has been preserved. You keep it in the drawer, and every now and then it opens and closes. Maybe, when I see children or pregnant women, then I smile and say, “What a beautiful belly you have,” and a veil of sadness comes over you. (Beatrice, 56 yrs)

[Getting married with the awareness that we could not have children meant that] the dream would not have been completed …. More than anything else, the idea of the future, of not being parents in future. At that time, we were happy as two but when we would have been 40 or 50 years old we would have missed having a child. In addition to my desire to become mother, which I missed. (Clara, 37 yrs)

Despite the disappointment about the impossibility of having a child, some of the participants did not consider the alternative of adoption because, although they claimed to be able to give the same affection to another child, the experience of the pregnancy, and of the child as the fruit of the love of two people, would be missing. Others, however, were discouraged by the long procedure or by the awareness of their precarious medical situation. These women strongly hoped that one day the situation may change, and it was for this reason that they also did not consider methods that are more permanent, such as sterilization:

I was terrified. In fact, I had told both my mother and my father that if the doctor should come out and say, “Look, we opened, we have to remove the uterus,” I absolutely would not give them consent. I was terrified of this because I had the thought that maybe tomorrow, I could have children. (Maria, 40 yrs)

Hope was strengthened by the impossibility of predicting whether medical discoveries might allow pregnancy in future:

Let’s put it this way, like an exam that you have to pass, the pregnancy we will see, but at the end, I mean, until you get to that day when you have to take the exam, … you have to take this test, you never know how [medical progress] is going to turn out. (Sara, 24 yrs)

Many participants did not accept the limitations imposed by PAH and were willing to risk their lives and the lives of their fetuses in the hope of becoming mothers. However, the partners of these women did not support or encourage their will to have a child at all costs and made it explicit that having children was not a priority for them:

My partner said “No,” he absolutely did not want to try to have a child … I did not accept this because I said, “This is a very personal thing and I want to try”. (Anna, 49 yrs)

At the beginning, he was a little bit hurt; he was a little bit stunned, but then, at the end, he said, “Look, I married you, I did not marry having children. I’m fine with you. I prefer to spend my life with you rather than with another person whom I love less and who can give me children,” and at the end, he accepted it. (P17, 52 yrs, FC III)

Women who had a child in the period prior to diagnosis expressed a feeling of having been lucky to have the opportunity to experience pregnancy and to have become mothers before the onset of the disease:

I am very happy not to have known of, maybe not to have had, at that time, the disease … because, certainly, the possibility of not having children becomes a very heavy thing; one thing is a personal choice, another thing is to feel like you cannot have them or at least discourage pregnancy. (Simona, 56 yrs)

## Discussion

**Fig. 1:**
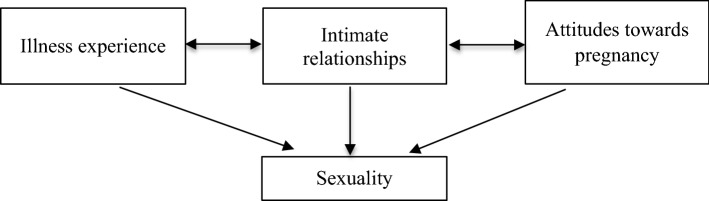
Map of the relationships between themes

The results of the present study have shown that the experience of sexuality of women with PAH depends on their experiences of illness, intimate relationships, and attitude toward pregnancy. Each of these themes are related to one another, as shown in Fig. 1 and illustrated below.

Participants’ narrations confirmed that the illness trajectory, in the case of PAH, is difficult from its very beginning. As it is a rare disease, recognition of its symptoms and reaching a diagnosis often takes time and involves frustration for the person who is ill (Galié et al., 2016; Kingman et al., 2014). When the diagnosis finally arrived, the participants usually experienced shock, shame, anger, and fear for the future (Guillevin et al., 2013). Moreover, this study’s participants often perceived the communication of the diagnosis to be detached and this made them experience the moment of their illness’ discovery as even more traumatic. After the first period of adjustment, the participants reported an improvement in their life that may be understood as being due to the reorganization of their life and the social support they received, which both contributed to fostering the resilience needed to cope with daily difficulties (Banerjee et al., 2018).

The illness was perceived to be so pervasive as to require a general reorganization of the women’s lives to distribute their energies optimally and establish new priorities. The tiredness and great fatigue, of which people with PAH complain, often prevented them from doing what they would have liked to do. Understanding their limits and finding strategies to cope with them may help these people to feel more satisfied with their lives (Bussotti et al., 2016; McKenna et al., 2006). This was the case for participants who described the disease as a companion in life rather than an enemy.

In line with previous studies (Bussotti & Sommaruga, 2018), some women reported that the fact that the symptoms of their pathology were not externally visible often led people to minimize their complaints or to consider them the result of fantasy or exaggeration. Group support, therefore, proves to be crucial for them (Galié et al., 2016) because discussing the symptoms with someone who is on the same journey is a source of physical and emotional support that provides information about the disease and instills hope and understanding in people suffering from chronic illnesses (Cipolletta et al., 2018, 2019).

The results of the present study also show a drastic closure to new social relationships, probably due to the fear of rejection, the shame associated with the role of a sick person, and a feeling of inadequacy in the relationship due to the sacrifices PAH could imply for other people. In contrast with this view, other participants in this study did not identify themselves with the illness but gave it a new meaning and reorganized their lives and relationships accordingly. As previously pointed out by Cipolletta et al. (2012, 2017a, 2017b), this approach to illness is usually associated with a better illness trajectory.

The various reactions of women with PAH could also be justified by their different ages and illness severity and, therefore, by the different difficulties they had encountered so far (McKenna et al., 2006). The women who chose to step away from relationships were older and had worse symptoms than those who turned to others to accept their illness.

A novel result of the present study concerns the sense of femininity of women with PAH. As with other aspects of their lives, femininity also underwent changes in varying ways according to the reorganization of these women’s lives, their illness experience, and body perception. A key aspect of sexual health for women is comfort with their bodies during sex because bodies are deeply involved in sexual encounters. They may be partially or fully naked and feel exposed in ways that are different from other areas of daily life. For these reasons, body image concerns play an important role in sexual pleasure and sexual functioning (Curtin et al., 2011). Chronic illnesses involve a shift from an initial state of embodiment, one in which the body is largely taken for granted in the normal course of everyday life, to an oscillation between states of disembodiment (i.e., embodiment in a dysfunctional state) and attempts at re-embodiment; the latter involving considerable biographical work on the part of the individual, which fundamentally transforms previous concepts of body, self, and society (Williams, 1996).

The results of the present study differentiated the participants’ experiences of their femininity on the basis of their illness experiences. Women who identified themselves with the illness did not want to devote care to their appearance and avoided showing their body or felt ashamed of it. On the contrary, those who did not identify themselves with the illness could regain a new sense of femininity.

Hill et al. (2018) underlined that negative body image caused by medical devices and altered physical appearance due to edema and flushing may also affect the intimacy of people with PAH. The results of our study confirmed that physical symptoms and possible medical devices led to a reduction in the participants’ sexuality (Schönhofer et al., 2001; Studer et al., 2012). Fatigue (the predominant symptom of this pathology), as well as the possible presence of a nasal cannula for oxygen and an infusion pump for drugs, made sexual relationships more complicated. In line with Guillevin et al. (2013), some participants in the present study also reported their partner’s fear of hurting them during intercourse or possibly worsening their health condition.

Most participants described their partners (when present) as very understanding and protective and recounted a reorganization within the couple’s relationship in terms of sharing more of the family management and paying greater attention to the woman’s wellbeing, which might also have been augmented by the fear of losing her. The pathology in these cases has fostered closeness between the spouses. On the other hand, sometimes, the women’s or their partners’ excessive concerns about the disease caused difficulties in the relationship because they led the couple to be trapped by too many constraints and the woman to feel inadequate and dependent.

Communication about sexuality is scarce in the context of this illness as it is for cardiovascular disease (Steinke et al., 2015). Participants reported that medical staff usually did not give information about sexuality and neither did they ask for it. The women emphasized that sexuality was an intimate and delicate topic, often associated with shame, which led to them avoiding speaking about it despite the curiosity and doubts that accompany this aspect. This experience may be interpreted in light of the taboo by which sexuality is often perceived (Haley et al., 1999), even more so in the context of the present research. Despite rapid change, sexual behavior in Italy seems, in many ways, to be anchored in the past, particularly for women, older people, practicing Catholics, and the less educated (Caltabiano & Dalla-Zuanna, 2013).

In Italy, there are no sex-education programs in schools, nor are there any legally recognized forms of homosexual unions. Italian women have sexual intercourse later and with fewer partners than their peers in other European countries. An international study (Currie et al., 2008) showed that the percentage of Italian girls who have sexual intercourse at age 16 (22%) is lower than that of their peers in Denmark (40%), in Sweden (32%), and in England (31%). In another study (Bajos & Bozon, 2008) only 15% of 26-year-old Italian women reported having had more than six sexual partners during their lifetime, compared to 25% of French women of the same age.

The results of a recent study (Caltabiano & Dalla-Zuanna, 2021) suggest that in the last few years an important step has been taken toward “the end of Catholic sexuality” in Italy. However, it would be wrong to conclude that, in the Italian context, the Church’s views on sexuality have become entirely irrelevant. There remain several behaviors and opinions where differences persist between the more and less religious, particularly for Italian women. Specifically, young Catholic women are less oriented toward hedonistic sexuality, especially outside stable couple relationships. Among the participants of the present study, religiosity did not have an impact on sexual health, except for a woman who considered sexuality inseparable from the possibility of having a child. This was also the only case where contraception was considered as a limitation for sexuality. Here, the dilemma is between ethics and health (Duarte et al., 2013) because the desire for pregnancy contrasts with the need to resort to contraception and, eventually, to abortion to preserve one’s own life.

With regard to the risks of pregnancy, this consequence of PAH was experienced as a limitation of the participants’ freedom and lives, generating feelings of anger, injustice, frustration, guilt, and sadness. The results of previous studies with women who cannot have children (Luk & Loke, 2014) found similar emotions, whereas there are no previous data specifically on the reactions of people with PAH. Therefore, these should be further investigated in more comprehensive studies.

Some participants also reported that the physicians’ communication about the risk of pregnancy was hasty and careless, and this kind of experience did not allow them to explore and, consequently, accept this limitation in their life. On the contrary, other participants reported that they were given empathetic and respectful communication about the situation, making them feel welcomed and helping them to accept the news.

This study has shown that younger women hoped their situation might change and that medicine might one day find a solution to allow them to become mothers without excessive risk. Other women were willing to risk their lives to experience the joy of pregnancy and could not consider the alternative of adoption or sterilization. As in the couples with infertility (Luk & Loke, 2014), the role and closeness of the partners who showed affection to the women and accepted the impossibility of having children, even before the women themselves did so, also proved to be fundamental in helping the participants to accept the situation. Finally, the issue of pregnancy was not problematic for the women who already had children because they had already experienced maternity.

### Limitations

A limitation of this study is represented by the small number of participants. The rarity of the pathology, as well as the health complications often related to it, limited the possibility of recruiting more participants in the study. Moreover, recruitment only involved women in care at one Italian center, thus preventing us from gaining knowledge about the experiences of women with PAH from different centers and different cultural contexts. Future studies might fill this gap. Another important limitation is the lack of the partners’ perspectives on intimate relationships and sexuality. Future studies might involve the dyad and might investigate the experience of women having a pregnancy during the pathology, which was missing in the present study.

### Conclusion

The present study fulfils the need for a thorough understanding of such fundamental and delicate issues as the management of sexuality and pregnancy in women with PAH. The results highlighted that the participants’ responses to the changes in their intimate relationships due to PAH were varied. For many participants, sexuality took a backseat because the disease absorbed most of their lives. Many participants also manifested feelings of inadequacy and shame that limited their intimacy and compromised their body image. These feelings could also lead these women to limit their relationships, precluding them from entering into a loving relationship. On the contrary, the acceptance of the limitations imposed by PAH on the part of the women and their partners, together with the reorganization of everyday life and sense of femininity on the basis of new priorities and a sense of trust in the relationship and the future, allowed some couples to maintain a satisfying intimate relationship.

These results may inform tailored interventions based on more targeted and effective methods of communication, the involvement of the women who are ill and those close to them, and the promotion of a new approach to their relationship and sexual life by also limiting the fear of the unknown and the experience of feeling “lost” that often accompany people with PAH.
